# Laryngeal Papillomata in Children

**Published:** 1910-07-30

**Authors:** 


### Laryngology and Rhinology.

### LARYNGEAL PAPILLOMATA IN CHILDREN.

Papilloma is the commonest variety of new growth found within the larynx. In adults it generally occurs as a single outgrowth, situated, like most other innocent neoplasms, at the junction of the anterior and middle thirds of the glottis; this spot, which is the "seat of election" for innocent growths, marks the centre of the vocal cord, for the posterior third of the glottis is formed by the arytenoid cartilage, and is the site of the amplest vibration and maximum irritation. Irritation, however, can hardly account for the occurrence of papillomata in children, the etiology of which is at present unexplained. They usually appear between the ages of twelve months and five years; sometimes they are cured by a single removal, but more often they show a persistent tendency to recur until the age of puberty, when they cease to re-form and may even disappear spontaneously. A single papilloma is uncommon in a child; on the contrary, they are often very numerous and grow from any part of the interior of the larynx, even from the posterior commissure, which is usually avoided by innocent tumours. They may completely fill the lumen of the larynx with a cauliflower-like mass, often grow from the subglottic region, and may even extend to the bifurcation of the trachea.

The earliest symptom is impairment of the voice. It should be remembered that long-continued hoarseness in a child, unaccompanied by constitutional disturbance, is nearly always due to laryngeal papillomata. The other, and very important symptom, dyspnœa, follows sooner or later; the narrowing of the laryngeal lumen is gradual, but, as in all forms of laryngeal obstruction in children, spasm is an important factor, and the dyspnœa is therefore variable and subject to exacerbations.

Laryngeal diagnosis is never a very easy matter in small children, but the symptoms just described afford strong presumptive evidence of the presence of papillomata. A small foreign body impacted in the larynx causes hoarseness with spasmodic dyspnœa, and may be entirely unsuspected by the parents, but the onset of these symptoms is sudden, and there is far more discomfort and cough. Diphtheria, membranous and spasmodic laryngitis can be distinguished by the constitutional disturbance and the shorter history; in laryngismus stridulus there is no hoarseness or any symptom between the attacks of spasm. The only other causes of chronic hoarseness in a child without signs of constitutional disease are the rare congenital abnormalities, such as a web, a malformation, or a nodule on the cord. The diagnosis can, of course, only be made absolute by inspection; a rapid glance is generally sufficient, and is not very difficult with a tractable child of five or over. It is nearly always possible with younger children if they are firmly secured by a blanket on the nurse's lap and a curved spatula used to pull forward the tongue. If this cannot be done, examination must be performed under anæsthesia, and may then be combined with treatment.

Before the introduction of the direct method of laryngoscopy the management of these cases presented the greatest difficulty. Removal by thyrotomy has been performed, but is considered unjustifiable by the highest authorities, for it is an operation not without danger; it carries a distinct risk of injury to the voice and, in view of the tendency to recurrence, it offers no prospect of radical cure. Tracheotomy must be performed if the dyspnœa has become urgent, and cases have been recorded in which the growths have disappeared after this operation; this, however, they very often fail to do, and intra-laryngeal removal becomes necessary. Intra-laryngeal removal is hardly possible before the age of six, but it is advisable to remove the growths as soon as possible, for the prolonged wearing of a tracheotomy tube involves decided risks of pulmonary disease as well as of damage to the trachea. It was, and perhaps it still is, the best method with children over six or seven to train the patient by repeated manipulation until removal becomes possible under inspection with the laryngeal mirror, for when once this has been accomplished it can be repeated as often as recurrence renders it necessary. For intractable children the combined cocaine and chloroform anæsthesia remained the only alternative. The method was not without danger, for the chloroform had to be given to a full degree in the sitting posture, and a fairly free use of cocaine was essential. Now, however, the method of direct laryngoscopy has revolutionised the treatment of these difficult cases. The speculum is passed under chloroform with the child in the left lateral position, a little cocaine and adrenalin applied to diminish spasm and secretion, and the growths removed with straight forceps under inspection. In severe cases two sittings are sometimes necessary to ensure complete removal, and the subglottic region and trachea should also be examined. In this way the growths can be removed from the youngest child, and tracheotomy is only required if they have been allowed to attain such a size as to cause severe dyspnœa before the case has been brought up for treatment. Recurrences are still to be expected, and the parents must be warned to bring the child up immediately on the return of any hoarseness.

